# Purified *Pyropia yezoensis* Pigment Extract-Based Tandem Dye Synthesis

**DOI:** 10.3390/md22050197

**Published:** 2024-04-25

**Authors:** Hojun Lee, Taejun Han, Jihae Park

**Affiliations:** 1 Bio Environmental Science and Technology (BEST) Lab, Ghent University Global Campus, 119-5, Songdomunhwa-ro, Incheon 21985, Republic of Korea; 2 Department of Animal Sciences and Aquatic Ecology, Ghent University, Coupure Links 653-Block F, B-9000 Ghent, Belgium; 3 Centre for Environmental and Energy Research, Ghent University Global Campus, 119-5, Songdomunhwa-ro, Incheon 21985, Republic of Korea

**Keywords:** extraction, phycoerythrin, purification, *Pyropia yezoensis*, tandem dye synthesis

## Abstract

Red phycoerythrin (R-PE) is a highly valuable protein found in an edible seaweed, *Pyropia yezoensis*. It is used extensively in biotechnological applications due to its strong fluorescence and stability in diverse environments. However, the current methods for extracting and purifying R-PE are costly and unsustainable. The aim of the present study was to enhance the financial viability of the process by improving the extraction and purification of R-PE from dried *P. yezoensis* and to further enhance R-PE value by incorporating it into a tandem dye for molecular biology applications. A combination of ultrafiltration, ion exchange chromatography, and gel filtration yielded concentrated (1 mg·mL^–1^) R-PE at 99% purity. Using purified PE and Cyanine5 (Cy5), an organic tandem dye, phycoerythrin-Cy5 (PE-Cy5), was subsequently established. In comparison to a commercially available tandem dye, PE-Cy5 exhibited 202.3% stronger fluorescence, rendering it suitable for imaging and analyzes that require high sensitivity, enhanced signal-to-noise ratio, broad dynamic range, or shorter exposure times to minimize potential damage to samples. The techno-economic analysis confirmed the financial feasibility of the innovative technique for the extraction and purification of R-PE and PE-Cy5 production.

## 1. Introduction

*Pyropia yezoensis* (Ueda), a marine red alga belonging to the genus *Pyropia*, family Bangiaceae, is an economically important marine crop. China, Japan, and Korea collectively produce 2,984,123 tonnes of *P. yezoensis* annually, with an additional 450 tonnes harvested from the wild [1]. *P. yezoensis* is used widely in Asian cuisine, and recent studies have demonstrated its cosmeceutical and pharmacological properties [2,3,4,5,6]. Approximately 40% of the soluble proteins in *P. yezoensis* are water-soluble fluorescent phycobiliproteins. Phycobiliproteins are categorized into three types: blue-green allophycocyanin, blue phycocyanin, and red phycoerythrin (R-PE). The pigments are used as natural coloring agents for food, healthy beverages, and cosmetics [7,8,9,10] and also possess anti-inflammatory, antioxidative, and anticancer properties [11,12,13,14]. 

R-PE is the primary light-harvesting pigment in red seaweed. It consists of a series of bilin chromophores (prosthetic groups) bound to hexamer (αβ)_6_γ-subunits made up of polypeptide chains (α, β, and γ) (Protein Data Bank: 1EYX) [15]. R-PE absorbs light in the blue-green region of the spectrum with absorption maxima at 498 nm and 565 nm and exhibits a fluorescence emission maximum at 580 nm [16]. Due to its high molar absorption coefficient of approximately 1.96 × 10^6^ M^–1^·cm^–1^ [17], thermal stability at temperatures up to 45 °C [17,18], and emission quantum yield (Φ) of ≥80% [19], R-PE has a diverse range of applications. It is used as a dye in food [20], as a photosensitiser in photodynamic therapy [21], and, more recently, in solar cells [22,23] and optically active sites within innovative liquid-based luminescent solar concentrators [24,25]. Furthermore, using R-PE as a fluorescent dye offers several advantages over traditional fluorescent dyes, with the potential improvement in current visualization techniques. Such advantages are attributed to its high extinction coefficient, broad excitation spectrum, narrow emission spectrum, and enhanced photostability [26,27]. In diagnostic and biomedical research, R-PE is employed extensively as a tracer in fluorescence immunoassays and microscopy. R-PE is 10–20 times brighter than the best conventional organic dyes and is considered the brightest natural fluorophore [28]. In research and clinical diagnostics, R-PE is often conjugated with other biomolecules, such as antibodies, to target and identify specific cells or biomolecules. It is also used as a fluorescent label to visualize and study molecular interactions, cellular signaling, and protein–protein interactions [29]. 

Several conventional methods have been employed to extract and purify R-PE from *P. yezoensis*, each with its unique advantages (Table 1). Such methods include ammonium sulfate precipitation, diethylethanolamine (DEAE) fast-flow column chromatography [30], hydroxyapatite column chromatography [31], Q Sepharose™ column chromatography [32], and liquid–liquid extraction [33,34]. The sustainability of the current extraction process is limited by its high implementation and operational costs and substantial energy requirements [35]; extraction costs constitute up to 60% of the total cost of R-PE [36]. Further purification may also be necessary for various intended end uses, such as food, feed, nutraceuticals, or cosmetics. The requirement could increase the final product price a hundred-fold [37]. Considering its high commercial value and potential medical applications, there is an urgent need for a cost-effective, efficient extraction protocol for obtaining high-purity R-PE. 

Staple crops can be transformed into high-value-added products via various strategies, including diversification (such as utilizing the crop for food, animal feed, and industrial products), improving quality (by enhancing the nutritional value), as well as optimizing and innovating processing methods. Such approaches enhance the market competitiveness of the crop, stimulate greater demand, and ultimately increase its value [38]. The aim of the present study was to enhance the value of *P. yezoensis* by developing an improved extraction and purification method to obtain high-purity R-PE. In addition, the potential application of the purified R-PE in fluorescence imaging analysis was investigated by developing a tandem dye using R-PE as a donor molecule and Cyanine5 (Cy5) as an acceptor molecule for Förster (fluorescence) resonance energy transfer. Finally, a techno-economic analysis (TEA) was performed to assess the economic feasibility and viability of the newly developed production process.

## 2. Results

### 2.1. Purification of Phycoerythrin

**Figure 1:**
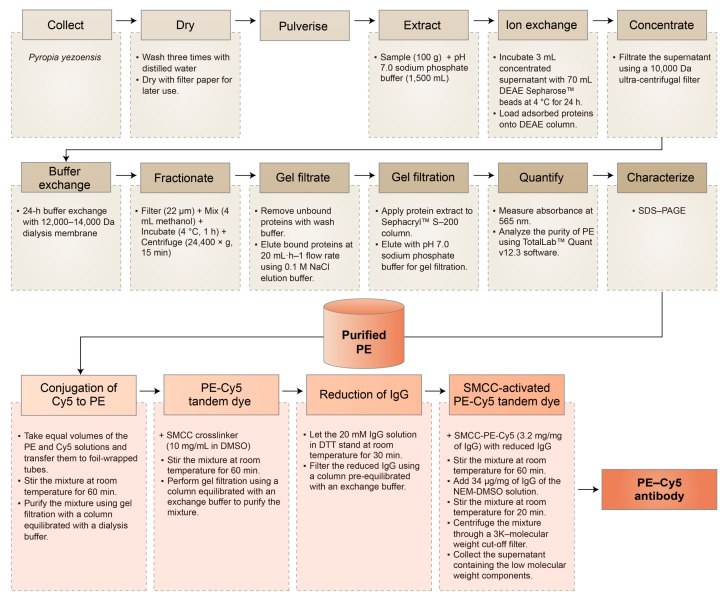
Schematic representation of the red phycoerythrin (R-PE) extraction and purification process (top) and combination of R-PE with Cyanine5 (Cy5) to synthesize the tandem dye, PE-Cy5 (bottom). SMCC, succinimidyl-4-(N-maleimidomethyl) cyclohexane-1-carboxylate; DMSO, dimethyl sulphoxide; IgG, immunoglobin G; DTT, dithiothreitol; NEM, N-ethylmaleimide.

**Figure 2:**
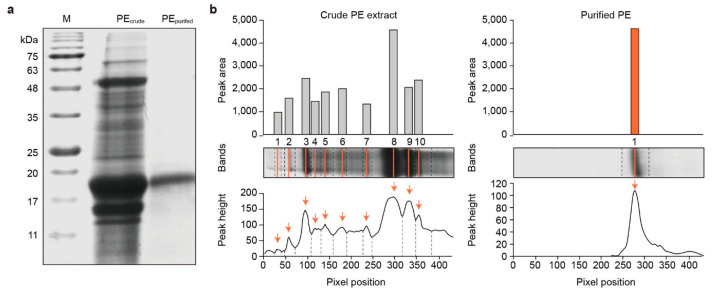
Analysis of crude and three-step-purified red phycoerythrin (R-PE) extracts from *Pyropia yezoensis*. (**a**) Sodium dodecyl sulfate-polyacrylamide gel electrophoresis of crude and purified R-PE extracts. Lane M, protein marker; Lane PE_crude_, crude protein extract; Lane PE_purified_, three-step-purified R-PE; (**b**) High-performance liquid chromatography analysis to determine the purity of R-PE in the extracts before (**left**) and after (**right**) the three-step separation process.

A three-step protein extraction and purification process was developed in the present study (Figure 1). Briefly, ionizable proteins that fall within the same size range as R-PE were extracted from 100 g of *P. yezoensis* using ultrafiltration. The method delivered R-PE with a concentration of 3 mg·mL^–1^. Ion exchange and size-exclusion chromatography were then used to purify and concentrate R-PE further. The protein concentration after ion exchange was 2.30 mg·mL^–1^, and size-exclusion chromatography delivered 1 mg·mL^–1^ of purified R-PE. Extracts obtained after each step were evaluated using sodium dodecyl sulfate-polyacrylamide gel electrophoresis (SDS-PAGE) combined with Coomassie Blue Staining (Figure 2A). The results indicated that the process removed all other proteins, retaining only purified R-PE. To determine whether the purified R-PE contained trace proteins that were not identifiable visually via electrophoresis, samples were subjected to external testing using the noise band detection method with high-performance liquid chromatography (HPLC) and TotalLab™ Quant v12.3 (TotalLab, Gosforth, UK) (Figure 2B). The HPLC profile of the crude extract exhibited ten peaks, whereas only one peak at 10.8 min was observed in the purified R-PE following three steps of extraction and purification. This peak corresponded to 99% of the total protein in the purified sample, indicating that highly purified R-PE was obtained. The yield and purity of R-PE obtained using our protocol were compared to those obtained by other R-PE purification processes described in the literature (Table 1).

### 2.2. Development of a Tandem Dye with Purified Phycoerythrin and Cyanine5

**Figure 3:**
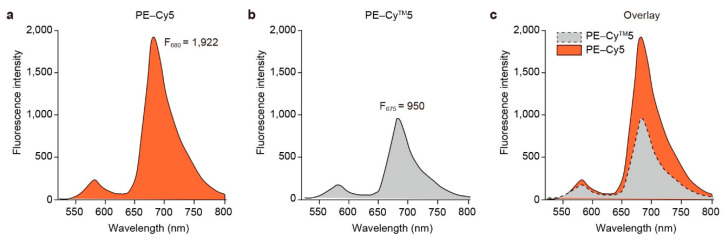
Fluorescence spectra of tandem dyes excited at a wavelength of 488 nm. (**a**) Emission spectrum at 680 nm for PE-Cy5, developed from purified red phycoerythrin (R-PE) extracted in the present study; (**b**) Emission spectrum at 675 nm for commercially available red phycoerythrin-cyanine5 (PE-Cy^TM^5) tandem dye; (**c**) Overlay of fluorescence spectra from PE-Cy5 and PE-Cy^TM^5.

The potential use of the purified R-PE in immunofluorescence or lab-on-a-chip assays was explored by developing PE-Cy5, a tandem dye utilizing R-PE and Cy5, based on a four-step conjugation protocol (Figure 1). Excitation of the final product by a 488 nm laser produced a strong emission signal at 680 nm, indicating that PE-Cy5 had been successfully synthesized (Figure 3A). Fluorescence, the most important property of fluorescent dyes, of the newly synthesized PE-Cy5 was compared with that of PE-Cy^TM^5, a commercially available tandem dye. PE-Cy^TM^5 exhibited a maximum fluorescence intensity of 950 at 675 nm (Figure 3B), whereas the same concentration of PE-Cy5 exhibited a maximum fluorescence intensity of 1922 at 680 nm (Figure 3A), representing an increase of 202.3% compared to PE-Cy^TM^5 (Figure 3C).

### 2.3. Economic Viability

**Figure 4:**
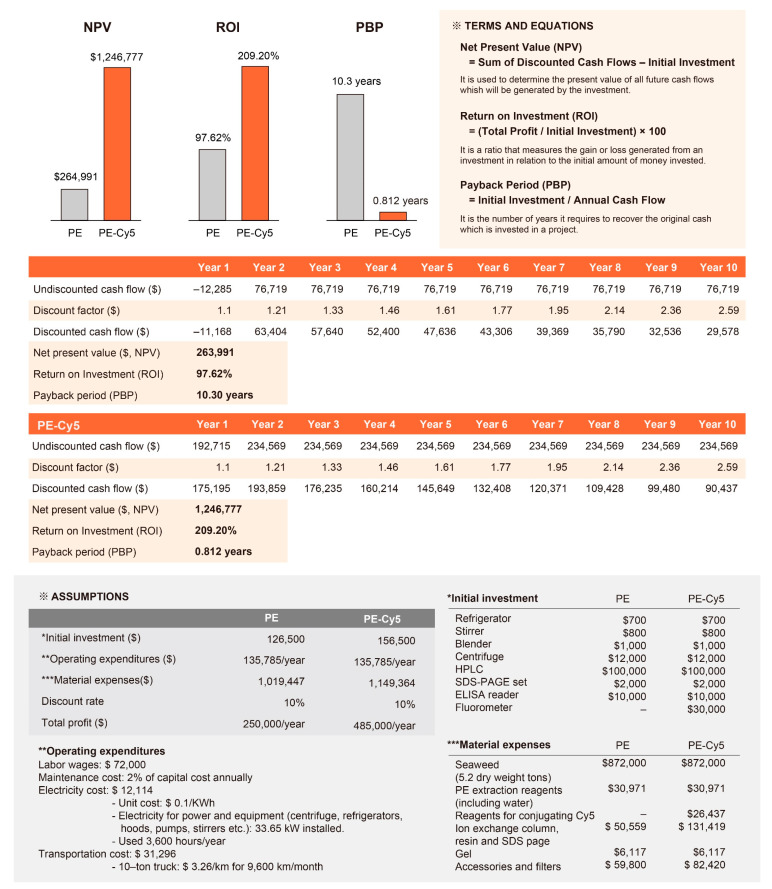
A techno-economic analysis of purified red phycoerythrin (R-PE) and its tandem dye, R-PE-Cyanine5 (PE-Cy5) production, based on an annual production target of 50 g for both PE and PE-Cy5.

The production of purified R-PE and PE-Cy5 was subjected to a comprehensive techno-economic analysis (TEA), a methodology for assessing the economic performance of a technology to evaluate its financial feasibility and investment potential (Figure 4). The initial investments required for the production of R-PE and PE-Cy5 were calculated as $126,500 and $156,500, respectively. The payback period (PBP) is used to calculate the number of years it would take to recover the initial investments via the generated net cash flow per year. The PBPs for the production of purified R-PE and PE-Cy5 were estimated to be approximately 10.3 years and 0.81 years, respectively. The return on investment (ROI) was calculated at approximately 97% for the production of purified R-PE and 209% for the production of PE-Cy5. The net present value (NPV) analysis was conducted based on a discount rate of 10%. The NPVs of both products were positive; for purified R-PE production, the NPV was approximately $263,991, while that of PE-Cy5 production amounted to $1,246,777. 

## 3. Discussion

Several methods have previously been developed for the extraction and purification of R-PE (Table 1) and typically involve a combination of organic solvent extraction, ammonium sulfate precipitation, and ion-exchange chromatography. However, despite their effectiveness, the methods have certain limitations [39]. The use of organic solvents can lead to protein denaturation and loss of function, while ammonium sulfate precipitation requires several time-consuming steps to achieve the desired purity [39]. Due to its fluorescence capacity and stability in various environments, R-PE is a highly desirable protein; however, its extraction and purification remain expensive and unsustainable. In the present study, a novel technique for the extraction and purification of R-PE from *P. yezoensis* was developed, and its economic viability was assessed. 

The newly developed R-PE extraction and purification method incorporates fractionation, ultrafiltration, ion exchange, and size-exclusion chromatography, which have not been used in combination prior to this study. During fractionation, differential centrifugation was used to isolate R-PE, thereby reducing the number of steps required to obtain pure protein samples. Ultrafiltration employs membrane filtration to separate proteins based on size and is more efficient than conventional ammonium sulfate precipitation [40,41]. Finally, ion exchange and size-exclusion chromatography are both highly specific and selective protein purification techniques that can reduce the loss of the desired protein during purification. Compared to those used in previously developed methods summarised in Table 1, the techniques offer the advantages of reduced purification time, improved protein yield and purity, as well as preservation of function. 

The high purity of the extracts was confirmed using SDS-PAGE and HPLC. To date, R-PE yields and concentrations have been calculated using absorbance values; however, the relatively simple approach can produce misleading results [26]. Therefore, a reliable quantification method for the comparison of R-PE yields is required. In the present study, protein bands from the extracts were analyzed. Subsequently, their purity was determined based on the size of the band corresponding to R-PE relative to that of the total lane instead of simply reporting the A_PE_/A_280_ ratio, where A_PE_ represents the maximum absorbance of R-PE. Furthermore, by utilizing an external testing center for analysis, protein purity was assessed objectively and accurately. Among the procedures used to extract R-PE from various organisms (Table 1), the method described in the present study achieved the highest yield of R-PE. 

R-PE is a naturally occurring protein, making it a sustainable and environmentally friendly alternative to synthetic dyes. The four-step conjugation protocol employed in the present study represents a significant advancement in the field of fluorescent dye synthesis. Using this protocol, we successfully synthesized the tandem PE-Cy5 dye, which exhibited a higher fluorescence intensity (202.3%) compared to a commercially available fluorescent dye (PE-Cy^TM^5). Therefore, PE-Cy5 can improve the sensitivity and accuracy of immunofluorescence and lab-on-a-chip assays [42]. Furthermore, the four-step conjugation protocol could be applied in the synthesis of other tandem dyes, facilitating the development of a new generation of highly fluorescent and photostable dyes for a diverse range of applications. The limited public information on the source of the phycoerythrin (PE) used in commercial tandem dyes, and the details of the conjugation process make it difficult for us to accurately distinguish the differences between PE-Cy5 (synthesized in this study) and PE-Cy^TM^5 (a commercial dye). However, given that PE is primarily derived from cyanobacteria and red algae, and that PE properties can vary between species, the differences in fluorescence intensity observed between PE-Cy5 and PE-Cy^TM^5 may be due to species-specific differences in PE and variations in the conjugation process including optimization of conjugation conditions, purity and stability of the conjugate, appropriate dye-protein ratio and buffer composition.

Currently, the market value of harvested and dried *P. yezoensis* is a modest $100–200 per kg [43]. However, the market value of this red seaweed can be increased considerably by expanding its applications beyond culinary uses [12]. With the approach proposed in the present study, 10 mg of purified R-PE can be obtained from 1 kg of *P. yezoensis*, increasing its market value to $1000–5000. By incorporating R-PE into PE-Cy5, this potential increase in value can be extended to several thousand to tens of thousands of dollars of scientific and commercial potential. The significant increase in value of purified R-PE derived from *P. yezoensis* can be attributed to several factors. Firstly, the process of obtaining purified R-PE involves complex extraction, purification and processing steps that add value to the final product. Secondly, R-PE is a valuable biochemical compound with a wide range of applications, particularly as a fluorescent marker in biological research and medical diagnostics. This increase in value reflects both the high demand for this compound and its wide range of applications. The high increase in value is reflected in the market trend as the global market demand for R-PE has been estimated at $2.3 billion in 2022, with a projected compound annual growth rate (CAGR) of 3.4% to 2032 [44]. The current market price of purified R-PE is approximately $495 per 1 mg (molecular weight of 240,000 g/mol; Cat No. B2011481; Molecular Depot LLC., San Diego, CA, USA), highlighting its significant economic value and widespread applications in science and industry. This underlines the economic importance of the methods used to extract and purify it.

To predict the economic and technical feasibility, viability, and sustainability of the process, the authors conducted a TEA. The ROIs of both R-PE and PE-Cy5 indicate high profitability, underscoring the significant potential for financial gains via the production of both products. Similarly, the positive NPV values indicate that the discounted cash inflows throughout the project’s timespan outweigh the initial investment and associated costs, highlighting the financial viability and attractiveness of investing in both purified R-PE and PE-Cy5 production. Together, the results provide compelling evidence for the financial viability of the products. However, it is essential to note that the analysis is based on certain assumptions that rely on the accuracy and completeness of the data provided. It will be crucial to conduct a comprehensive evaluation that includes factors such as market demand, competition, operational considerations, and potential risks, to enable prospective investors and stakeholders to make informed investment decisions. 

In summary, this study presents a novel approach for the extraction and purification of R-PE from *P. yezoensis*, with notable implications for various industries. The three-step method offers advantages over previously used protocols in terms of yield, efficiency, and preservation of protein function. The synthesized tandem dye, PE-Cy5, demonstrates superior fluorescence properties over commercially available dyes, promising advancements in visualization techniques. Our economic analysis revealed the high profitability and financial viability of both R-PE and PE-Cy5 production, providing strong support for their commercial potential.

**Table 1:** Summary of red phycoerythrin (R-PE) extraction and purification methods reported in the literature.

Species	R-PE Extraction and Purification Steps	PE Extraction (mg·g^−1^)	Purity Index (A_565_/A_280_)	Reference
*Amphiroa anceps*	Extract PBPs from fresh algae using 50 mM PBS (pH 7.4) with 150 mM NaCl, NaN_3_, 2-mercaptoethanol, and EDTA. Extract biliproteins using (NH_4_)_2_SO_4_ and perform ion-exchange chromatography on R-PE samples using a Q Sepharose™ Fast Flow column. Elute red-colored fractions using linear gradient elution with NaCl and monitor at wavelength 280 nm. Purify R-PE using gel filtration chromatography with Sepharose™ CL-6B and fast protein liquid chromatography. Collect bright-red fractions for characterization.	0.71	*NA*	Kawsar et al. [45]
*Gracilaria gracilis*	Homogenize algae in liquid nitrogen, suspended in phosphate buffer (20 mM, pH 7.1), extract at a 1:20 ratio, centrifuge, and collect supernatant as the crude extract. Apply the crude extract on a DEAE Sepharose™ Fast Flow column, perform elution using 200 mM NaCl, adjust the flow rate to 4 mL·min^–1^, and collect the fractions containing R-PE.	0.24	3.25	Nguyen et al. [46]
*Gracilaria lemaneiformis*	Cut *G. lemaneiformis* and lyse cells with hypotonicity and break further with a refiner, filter, and centrifuge. Precipitate with 65% saturated ammonium sulfate to obtain a crude sample for centrifugal partition chromatography separation. Fill the upper and lower centrifugal partition chromatography channels with 50% saturated ammonium sulfate solution. Load the sample into the inlet terminal of the water channel and elute with a linear gradient of ammonium sulfate solution and water at designated flow rates. Collect eluents and analyze absorbance values. Record the absorption spectrum of purified R-PE using a UV-visible spectrophotometer with a scanned wavelength from 250 to 700 nm. For native PAGE analysis, mix the sample with native PAGE sample buffer and deionized water. Analyze on 3–12% Bis-Tris protein gel and stain with CBB R250. For SDS-PAGE analysis, mix concentrated samples with SDS protein gel loading solution, heat, analyze on NuPAGE™ 4–12% Bis-Tris protein gel, and stain with CBB R250.	5	6.5	Gu et al. [47]
*Gracilariatenuistipitata*	Homogenize 1 kg of *G. tenuistipitata* in distilled water and freeze–thaw twice. Filter the mixture to remove the cell residue. Add 0.2 M ammonium sulfate to the crude extract and incubate at 4 °C overnight. Filter the crude extract to remove the precipitate. Determine the R-PE quantity using empirical formulae. Isolate R-PE using a STREAMLINE™ column filled with Phenyl Sepharose™ 6 Fast Flow. Wash the column with 0.5 M ammonium sulfate and elute with three different concentrations (0.5, 0.2, and 0.05 M) of ammonium sulfate. Collect the fractions containing PBPs and record the absorption spectrum. Dialyze the eluate against different concentrations of ammonium sulfate. Purify the dialyzed eluate using ion-exchange chromatography on a DEAE Sepharose™ column. Load the fractions separately onto the column and elute with 10 mM sodium acetate at a pH gradient from 4.5 to 4.0, to collect R-PE.	0.03	>4	Zhao et al. [48]
*Heterosiphonia japonica*	Extract PBPs with 50 mM phosphate buffer, NaN_3_, 2-mercaptoethanol, and EDTA. Salt out biliproteins with (NH_4_)_2_SO_4_ at 85% saturation and collect the R-PE extract. Purify R-PE via gel filtration with Sepharose™ CL-4B and Sephadex™ G-200, and perform ion-exchange chromatography on a DEAE Sepharose™ Fast Flow column. Evaluate R-PE purity via native PAGE in neutral and alkaline buffer systems.	*NA*	4.89	Sun et al. [49]
*Mastocarpus stellatus*	Prepare seaweed extract by homogenizing dry algal powder in phosphate buffer (20 mM, pH 7.1); centrifuge the extract and repeat the process four times to maximize yield. Fractionate the crude extract with ammonium sulfate, recover the precipitate via centrifugation, and dissolve it in phosphate buffer, followed by dialysis overnight. Apply the extract on an anion-exchange column and develop elution with a three-step increase in buffer ionic strength. Collect fractions containing R-PE at 200 mM NaCl using a high-performance liquid chromatography system with two buffers and a diode array detector.	0.11	1.91	Nguyen et al. [50]
*Michrochaete*	Harvest cyanobacterial biomass via centrifugation and wash with distilled water. Dry overnight at 50 °C. Extract PE in 0.1 M potassium phosphate buffer (pH 7.1) by repeated freezing and thawing. Record absorbance. Optimise PE extraction using 0.1 M acetate buffer (pH 6.0). Add solid ammonium sulfate to the crude extract to achieve 65% saturation. Allow the solution to stand for 12 h and then centrifuge. Resuspend pellets in a small volume of 50 mM acetic acid-sodium acetate buffer (pH 7.1) and dialyze overnight against the same buffer. Apply the dialyzed solution to a pre-equilibrated column of DEAE-cellulose in 50 mM acetate buffer (pH 7.1) at a flow rate of 0.5 mL·min^–1^. Collect pink-colored elutes and analyze each fraction using absorption spectroscopy to characterize PE. Calculate the purity of PE at each step in terms of the A_555_/A_280_ ratio.	0.198 (mg·mL^–1^)	4.1 (A_555_/A_280_)	Afreen and Fatma [51]
*Polysiphonia urceolata*	Mix frozen *P. urceolata* with distilled water and allow cell lysis. Filter and centrifuge the supernatant. Add solid ammonium sulfate to achieve 0.50 M concentration. Load the extract onto a STREAMLINE™ column with Phenyl Sepharose™ and wash with 0.50 M ammonium sulfate. Elute with 0.20, 0.10, and 0.05 M ammonium sulfate solutions and distilled water successively. Apply eluates onto an ion-exchange column with Q Sepharose™ and wash with 0.05 M NaAc and 0.05 M phosphate buffer (pH 7.0). Elute with NaCl gradient from 0 to 0.30 M in 0.05 M phosphate buffer (pH 7.0). Measure the volumes and optical densities of all eluates to determine the quantity and purity of R-PE.	0.40	3.26	Niu et al. [31]
*Polysiphonia urceolata*	Thaw frozen algae in 0.02 M Na-phosphate buffer (pH 7.0), filter, and centrifuge to obtain the PE-containing supernatant. Fractionate supernatant with ammonium sulfate at 25% and 45% (*w*/*v*), collect red supernatant and precipitate, dissolve precipitate in 20 mM phosphate buffer (pH 7.0), and dialyze overnight. Apply dialyzed R-PE samples on DEAE Sepharose™ Fast Flow column pre-equilibrated with 20 mM phosphate buffer (pH 5.6) containing 0.05 M NaCl. Wash the column with the same buffer, elute with 20 mM phosphate buffer containing 0.05 M NaCl with a pH gradient (pH 5.6–4.0, 2 × 50 mL) at 1 mL·min^−1^, monitor eluate at 280 nm, and collect 2-mL fractions. Perform native PAGE and SDS-PAGE using the Bio-Rad vertical slab discontinuous gel electrophoresis apparatus with 5% stacking gel as well as 7.5% and 15% separating gel, respectively. Stain gels with CBB R250.	1.79 (ca.)	5.6	Liu et al. [52]
*Pyropia yezoensis*	Homogenize 50 g dried *P. yezoensis* in 250 mL PBSE (pH 6.8) with EDTA, centrifuge at 8500× *g* and 4 °C for 15 min. Precipitate supernatant with 20% ammonium sulfate and discard the pellet. Precipitate the supernatant again with 50% ammonium sulfate and dissolve the pellet in PBSE. Precipitate the solution with 10% ammonium sulfate, collect the supernatant, precipitate again with 40% ammonium sulfate, and discard the supernatant. Dissolve the final pellet in 50 mM PBSE and centrifuge at 19,000× *g* and 4 °C for 30 min. Desalt with Sephadex™ G-25 and purify with hydroxyapatite. Perform gradient elution with PBSE at different concentrations to collect the PE-containing eluent and purify it with regenerated hydroxyapatite. Analyze PE purity and content via protein absorption, SDS-PAGE (14% separating and 5% stacking gel), and native PAGE (5% separating and 5% stacking gel). Determine purity based on the A_565_/A_280_ ratio.	*NA*	5.5	Cai et al. [53]
*Pyropia yezoensis*	Chop and immerse 200 g *Py. yezoensis* in PBSE buffer (50 mmol·L^−1^ sodium phosphate and 1 mmol·L^−1^ EDTA) at a ratio of 1:5 g·mL^−1^. Homogenise and centrifuge the mixture at 10,000× *g* and 4 °C for 15 min. Collect 980 mL supernatant and precipitate 420 mL with ammonium sulfate using seven gradients of three parts/concentration × 20 mL/part. Collect the supernatant and dissolve the pellet in PBSE after overnight precipitation and centrifugation. Measure solution spectra at each step with an Ultrospec 2000 spectrophotometer to calculate PBP purity and content.	0.94	1.94	Cai et al. [54]
*Porphyra yezoensis*	Fragment 28 g of the leafy gametophyte of *P. yezoensis* in 300 mL 10 mM phosphate buffer (pH 6.8) using a triturator for 30 min, followed by freeze–thawing. Filter the slurry with gauze, repeat the procedure three times, and pool all supernatants. Add powdered (NH_4_)_2_SO_4_ to a final concentration of 0.50 M and maintain at 4 °C overnight. Centrifuge at 3000× *g* for 10 min and determine the quantity of R-PE. Fill STREAMLINE™ column with Phenyl Sepharose™ and equilibrate with 0.50 M ammonium sulfate solution. Apply the crude R-PE extract at room temperature and conduct the expanded bed run. Determine the quantity and purity of isolated R-PE in each eluate. Combine eluates and dialyze against MilliQ water. Pump into an anion-exchange column (20 × 1 cm) loaded with 15 mL DEAE Sepharose™ in a downward direction. Wash with 10 mM NaAc (pH 4.2) and develop with 50 mM phosphate buffer (pH 6.8) containing an increasing step gradient of NaCl (0–0.20 M) at a flow rate of 2.5 mL·min^−1^. Collect the red-colored eluate and record the UV-visible and fluorescence spectra at room temperature.	0.82	4.5	Niu et al. [55]
*Porphyra yezoensis*	Store freshly harvested *P. yezoensis* at −80 °C until use. Isolate PE from lyophilized *P. yezoensis* and measure the visible absorption spectrum of the fraction containing PE using a spectrophotometer. Use an extinction coefficient of 80.2 at 565 nm to quantify PE. Obtain phycoerythrobilin from the methanolysis of PE by heating at 90 °C for 3 h, followed by centrifugation and extraction from the chloroform layer. Use an extinction coefficient of 25,200 at 591 nm to quantify phycoerythrobilin. Analyze PE using an HPLC system with a photodiode array detector, using an ODS-80Ts column and a mobile phase of methanol:water:acetic acid (50:50:1, *v*:*v*:*v*) at a flow rate of 1.0 mL·min^–1^, with the column temperature maintained at 40 °C.	*NA*	*NA*	Sakai et al. [56]
*Portieria hornemannii*	Prepare *P. hornemannii* by adding 50 g of fresh thallus to 125 mL of 0.02 mM phosphate buffer at pH 7.2, then pulverize, filter, and repeatedly freeze–thaw. Centrifuge at 12,500× *g* for 20 min, precipitate with 35% and 55% saturated ammonium sulfate and dialyze against 0.05 M phosphate buffer at pH 7.2. Record levels of R-PE, phycocyanin, and allophycocyanin. Load the dialyzed sample onto a Q Sepharose™ anionic-exchange column (30 × 2.5 cm) and elute with increasing concentrations of NaCl. Collect R-PE-rich fractions, dialyze, concentrate, and store. Conduct native PAGE of R-PE using a Bio-Rad Electrophoresis Apparatus. Perform two-dimensional electrophoresis of R-PE and stain with silver nitrate.	0.86	5.21	Senthilkumar et al. [32]
Red algae	Homogenise algal powder in extraction buffer (1 g/20 mL ratio). Agitate system (150 rpm) for 20 min to 12 h. Centrifuge at 25,000× *g* for 30 min and recover supernatant. Perform multiple extractions if necessary. Pool all supernatants. Pre-equilibrate DEAE Sepharose™ Fast Flow column with buffer A (10-times column void volume). Load supernatant on top of the column without overloading. Rinse the column with buffer A at a flow rate of 4 mL·min^–1^. Elute R-PE with a three-step increase in buffer ionic strength: 150 mM, 200 mM, and 1 M NaCl, for 10 min each, at a flow rate of 4 mL·min^–1^. Monitor absorption at 280 and 565 nm. Collect fraction eluted with 200 mM NaCl. Desalt overnight via dialysis against phosphate buffer. Estimate the purity index of the fraction based on the A_565_/A_280_ ratio.	*NA*	*NA*	Dumay et al. [57]

A purity index of 3.2 is considered the standard for R-PE [58]. NA, not applicable; PBP, phycobiliprotein; PBS, phosphate-buffered saline; R-PE; R-phycoerythrin; DEAE, diethylethanolamine; PC, phycocyanin; APC, allophycocyanin; PAGE, polyacrylamide gel electrophoresis; CBB R250, Coomassie Brilliant Blue R250.

## 4. Materials and Methods

### 4.1. Sample Material

*P. yezoensis* was collected from aquaculture beds in Jangbong-do, Incheon, Korea. The algae were then washed three times with distilled water, dried using filter paper, and stored for 24 h at room temperature (23–25 °C).

### 4.2. Purification of Phycoerythrin 

#### 4.2.1. Fractionation and Ultrafiltration

Fractions containing proteins within the same size range as R-PE were obtained from dried seaweed using ultrafiltration. Briefly, in a blender, 100 g of dried *P. yezoensis* was crushed and mixed with 1500 mL of sodium phosphate buffer (pH 7.0; sodium phosphate dibasic heptahydrate [Na_2_HPO_4_·7H_2_O], ≥99.9%, CAS No. 7558-79-4; Sodium phosphate monobasic monohydrate [NaH_2_PO_4_·H_2_O], ≥99.5%, CAS No. 10049-21-5; Sigma-Aldrich, St. Louis, MO, USA). The mixture was incubated at 4 °C for 24 h with gentle stirring (98× *g*). Following centrifugation at 24,400× *g* for 15 min at 4 °C, the supernatant was collected and filtered through a 22-µm filter paper (Whatman plc, Buckinghamshire, UK) to remove any large impurities. The filtered supernatant (6 mL) was mixed with pure methanol (4 mL; ≥99.9%, CAS No. 67-56-1; Sigma-Aldrich) and incubated at 4 °C for 1 h, with gentle stirring, followed by centrifugation at 24,400× *g* for 15 min at 4 °C. A buffer exchange was then performed over the course of 24 h, using sodium phosphate buffer (pH 7.0) and a dialysis membrane with a molecular weight cut-off of 12,000–14,000 Da (Spectra/Por™ 2 RC Dialysis Membrane Tubing, Thermo Fisher Scientific, Waltham, MA, USA). Finally, the supernatant and filtrate were separated using an ultra-centrifugal filter with a molecular weight cut-off of 10,000 Da (Pierce™ Protein Concentrators PES, 10K MWCO, Thermo Fisher Scientific), and a highly concentrated protein mixture was obtained.

#### 4.2.2. Ion Exchange

Ion exchange was used to separate R-PE from other ionizable proteins. After fractionation and concentration, 3 ml of the concentrated supernatant was adsorbed onto 70 mL of DEAE Sepharose™ beads (Cytiva, Marlborough, UK) for 24 h at 4 °C while stirring to increase the adsorption strength. Beads with adsorbed proteins were then loaded onto a DEAE column (DFF100, Sigma-Aldrich). After removal of unbound proteins using wash buffer, the bound proteins were eluted from the column at a flow rate of 20 mL·h^–1^ using elution buffer supplemented with 0.1 M sodium chloride (NaCl, 99.9%, CAS No. 7647-14-5; Sigma-Aldrich). R-PE in the purified sample was subsequently quantified by measuring the absorbance at 565 nm using an enzyme-linked immunosorbent assay (ELISA) reader (Infinite 200 Pro, Tecan, Männedorf, Switzerland). 

#### 4.2.3. Gel Filtration

Following ion exchange, the protein extract was applied to a column containing Sephacryl™ S-200 beads (Cytiva). Since separation using gel filtration is not affected by buffer properties, elution was performed using sodium phosphate buffer (pH 7.0). To quantify R-PE, the absorbance of each eluted section was measured at a wavelength of 565 nm using an ELISA reader (Infinite 200 Pro, Tecan, Männedorf, Switzerland).

#### 4.2.4. Sodium Dodecyl Sulfate-Polyacrylamide Gel Electrophoresis

R-PE extracts from each of the extraction and purification steps were characterized using SDS-PAGE to determine the molecular mass of R-PE (Figure 2). SDS-PAGE was performed at room temperature in a vertical chamber using a 12.5% polyacrylamide gel containing 0.1% sodium dodecyl sulfate (SDS, ≥99.0%, CAS No. 151-21-3; Sigma-Aldrich), 4% acrylamide (≥99.0%, CAS No. 79-06-1; Sigma-Aldrich), and 0.1% bisacrylamide (≥99.5%, CAS No. 110-26-9; Sigma-Aldrich). Proteins were visualized via Coomassie Brilliant Blue staining (1610436, Bio-Rad, Hercules, CA, USA). The molecular mass of protein bands was determined by comparison using a molecular mass marker (Prestained Molecular Weight Marker, Sigma-Aldrich).

#### 4.2.5. Analyzes of Purified Proteins

The purity of R-PE obtained via the three-step purification process was determined using automated analysis using TotalLab™ Quant v12.3 software (TotalLab). An objective analysis of the purified proteins was also performed by the Korea Institute of Basic Science using HPLC (Agilent 1200 series with an Aeries WIDEPORE [3.6 µm] C4 column [150 × 4.6 mm]; Agilent Technologies, Santa Clara, CA, USA). The solvents used were 0.1% trifluoroacetic acid (≥99.0%, CAS No. 76-05-1; Sigma-Aldrich) in water and 0.075% trifluoroacetic acid in acetonitrile (≥99.9%, CAS No. 75-05-8; Sigma-Aldrich), with a flow rate of 1 mL·min^–1^ at a column temperature of 40 °C.

### 4.3. Development of a Tandem Dye with Purified R-PE and Cy5

To test the suitability of the purified R-PE for application in immunofluorescence or lab-on-a-chip assays, a tandem dye was developed using a four-step conjugation process, as described previously [59].

#### 4.3.1. Conjugation of Cy5 to R-PE

Purified R-PE (10 mg) was dissolved in 1 mL sodium phosphate buffer (pH 7.0), while 10 mg Cy5 (GC35769, GlpBio, Montclair, CA, USA), an organic dye, was dissolved in 1 mL anhydrous dimethyl sulfoxide (DMSO, ≥99.9%, CAS No. 67-68-5; Sigma-Aldrich). Equal volumes of both solutions were aliquoted into foil-wrapped tubes before being stirred at room temperature for 1 h. Conjugated proteins were purified from the reaction mixture using a gel filtration column (Sephadex G-100, Sigma-Aldrich) equilibrated with a dialysis buffer (50 mM disodium phosphate [Na_2_HPO_4_, ≥99.0%, CAS No. 7558-79-4; Sigma-Aldrich] and 1 mM ethylenediaminetetraacetic acid [EDTA, ≥99.0%, CAS No. 25102-12-9; Sigma-Aldrich], pH 7.0).

#### 4.3.2. Conjugation of Succinimidyl-4-(N-maleimidomethyl) Cyclohexane-1-Carboxylate (SMCC) to PE-Cy5

A 10-mg·mL^–1^ stock solution of SMCC (CAS No. 64987-85-5; Sigma-Aldrich) in anhydrous DMSO was prepared immediately before use. Thereafter, 11 µL SMCC per mg of PE-Cy5 was added to the foil-wrapped test tubes containing PE-Cy5, and the solution was incubated for 1 h at room temperature, with agitation. The obtained PE-Cy5 was subsequently passed through a gel filtration column (Sephadex G-100, Sigma-Aldrich) equilibrated with exchange buffer containing 50 mM 2-(N-morpholino) ethanesulfonic acid (MES, ≥99.0%, CAS No. 145224-94-8; Sigma-Aldrich) and 2 mM EDTA, pH 6.0.

#### 4.3.3. Reduction in Immunoglobulin G (IgG)

An IgG solution containing 2 mM dithiothreitol (DTT, ≥99.5%, CAS No. 3483-12-3; Sigma-Aldrich) was prepared by adding 20 µL of a 1 M DTT stock solution to 1 mL of IgG (I5381, Sigma-Aldrich) and incubated for 30 min at room temperature without further mixing to minimize the reoxidation of cysteine to cystine. The reduced IgG was then passed through a filtration column (Pierce™ Centrifuge Columns, Thermo Fisher Scientific) pre-equilibrated with an exchange buffer (50 mM MES and 2 mM EDTA, pH 6.0).

#### 4.3.4. Covalent Conjugation of Antibodies to SMCC-PE-Cy5

SMCC-PE-Cy5 (3.2 mg per mg of IgG) was added to a reaction tube containing reduced IgG, which was then covered with aluminum foil and rotated at room temperature for 1 h. A fresh solution of 10 mg N-ethylmaleimide (NEM, ≥99.0%, CAS No. 128-53-0; Sigma-Aldrich) was prepared in 1.0 mL anhydrous DMSO. Of the NEM-DMSO solution, 34 µg (3.4 µL) per mg of IgG was added to the reaction tube, and the tube was agitated at room temperature for 20 min. The final product was centrifuged through a 3,000-molecular weight cut-off filter, as instructed by the manufacturer (Pierce™ Protein Concentrators, PES, 3K MWCO, Thermo Fisher Scientific) to obtain two supernatants containing low- and high-molecular-weight proteins, respectively. After collection, the supernatant containing the low-molecular-weight components was placed in a fluorometer (Gemini EM plate reader, Molecular Devices, San Jose, CA, USA) alongside the commercially available dye, PE-Cy™5 (BD Biosciences, Franklin Lakes, NJ, USA). After excitation at a wavelength of 488 nm, the maximum fluorescence intensity of both dyes was measured.

#### 4.3.5. Techno-Economic Analysis

A TEA was conducted to evaluate the financial viability of the production process under investigation. The key metrics considered in the analysis were NPV, ROI, and PBP. The calculations were performed as shown in Figure 4.

## Data Availability

Data collected in this study is available from the corresponding author upon reasonable request.
